# Critical Role of NADPH Oxidase in Neuronal Oxidative Damage and Microglia Activation following Traumatic Brain Injury

**DOI:** 10.1371/journal.pone.0034504

**Published:** 2012-04-02

**Authors:** Quan-Guang Zhang, Melissa D. Laird, Dong Han, Khoi Nguyen, Erin Scott, Yan Dong, Krishnan M. Dhandapani, Darrell W. Brann

**Affiliations:** 1 Department of Neurology, Institute of Molecular Medicine and Genetics, Georgia Health Sciences University, Augusta, Georgia, United States of America; 2 Department of Neurosurgery, Georgia Health Sciences University, Augusta, Georgia, United States of America; University of North Dakota, United States of America

## Abstract

**Background:**

Oxidative stress is known to play an important role in the pathology of traumatic brain injury. Mitochondria are thought to be the major source of the damaging reactive oxygen species (ROS) following TBI. However, recent work has revealed that the membrane, via the enzyme NADPH oxidase can also generate the superoxide radical (O_2_^−^), and thereby potentially contribute to the oxidative stress following TBI. The current study thus addressed the potential role of NADPH oxidase in TBI.

**Methodology/Principal Findings:**

The results revealed that NADPH oxidase activity in the cerebral cortex and hippocampal CA1 region increases rapidly following controlled cortical impact in male mice, with an early peak at 1 h, followed by a secondary peak from 24–96 h after TBI. In situ localization using oxidized hydroethidine and the neuronal marker, NeuN, revealed that the O_2_^−^ induction occurred in neurons at 1 h after TBI. Pre- or post-treatment with the NADPH oxidase inhibitor, apocynin markedly inhibited microglial activation and oxidative stress damage. Apocynin also attenuated TBI-induction of the Alzheimer's disease proteins β-amyloid and amyloid precursor protein. Finally, both pre- and post-treatment of apocynin was also shown to induce significant neuroprotection against TBI. In addition, a NOX2-specific inhibitor, gp91ds-tat was also shown to exert neuroprotection against TBI.

**Conclusions/Significance:**

As a whole, the study demonstrates that NADPH oxidase activity and superoxide production exhibit a biphasic elevation in the hippocampus and cortex following TBI, which contributes significantly to the pathology of TBI via mediation of oxidative stress damage, microglial activation, and AD protein induction in the brain following TBI.

## Introduction

Traumatic brain injury (TBI) is the leading cause of death and disability in young people in the United States. Approximately 1.5 million people are affected each year with TBI, and despite advances in research and care, severe TBI still exhibits a mortality index as high as 35%–40%. Thus, the clinical outcome of severely head-injured patients is still poor. Primary damage in TBI is thought to result from mechanical forces applied to the skull and brain at the time of impact, leading to focal or diffuse brain injury patterns [1]. Secondary brain injuries, on the other hand, evolve over time [2]. These are characterized by a complex cascade of biochemical events that lead to neuroinflammation, brain edema, and delayed neuronal cell death [3]–[7].

Oxidative stress has been implicated to play a significant role in the pathology of TBI [8]–[10]. The most commonly occurring cellular free radical is superoxide radical (O_2_^−^), which is produced when an oxygen molecule gains one electron from another substance. Excess O_2_^−^ leads to tissue damage by promoting hydroxyl radical (OH^−^) formation through hydrogen peroxide (H_2_O_2_), and by combining with nitric oxide to form peroxynitrite (ONO_2_^−^), a powerful oxidant formed from superoxide and NO that can damage a wide array of molecules in cells [11]. It has been generally assumed that mitochondria are the major source of O_2_^−^ following brain injury [12]. However, recent work has shown that the enzyme, NADPH oxidase can also contribute to O_2_^−^ production in cells. NADPH oxidase is a membrane enzyme composed of several subunits that include NOX and phox subunits [13], [14]. There are several isoforms of NOX, termed NOX 1–5 [13]. Previous work by our laboratory and others has shown that the NOX2 NADPH oxidase is highly localized in the cerebral cortex and hippocampal CA1 region [15], [16]. Our laboratory and others also demonstrated that NOX2 is localized in neurons and microglia in the cortex and hippocampus [13], [15], [16]. NOX2 activation is dependent upon forming an active complex with several phox subunits (p47phox, p67phox, p40phox) and activated Rac1, which upon cell stress or activation translocate to the membrane from the cytoplasm to form the activated NADPH oxidase complex [13], [15]. Work from several labs including our own has shown that over-activation of neuronal NOX2 NADPH oxidase contributes significantly to ischemic oxidative damage to neurons and other cell types following cerebral ischemia [13], [16], [17]. NADPH oxidase is also localized in microglial and thus has the potential to contribute to neuroinflammation, as the extracellular ROS produced by microglia is directly toxic to neurons, and intracellular ROS in the microglia can amplify the production of several pro-inflammatory and neurotoxic cytokines [18].

Currently, there is little known on the role of NADPH oxidase in TBI. One recent report suggested that NADPH oxidase plays a role in microglia activation in the cerebral cortex at 24 h–48 h following TBI [19]. It is unknown whether NADPH oxidase mediates earlier elevations in ROS that occurs within 1–3 h after TBI, and whether it plays a significant role in oxidative stress induction and damage that has been shown to occur as early as 3 h after TBI [9]. It is also unclear as to whether NADPH oxidase may play a role in the induction of Alzheimer's disease (AD)-related proteins such as amyloid processing protein (APP) and β-amyloid, which have been implicated in the pathology of TBI. The current studies were designed to address these key deficits in our knowledge.

## Methods

### Controlled Cortical Impact

Animal studies were approved by the Committee on Animal Use for Research and Education at Georgia Health Sciences University (protocol # BR10-05-330), in compliance with NIH guidelines. Adult male CD-1 (Charles River, Wilmington, MA) mice were anesthetized with xylazine (8 mg/kg)/ketamine (60 mg/kg) and subjected to a sham injury or controlled cortical impact, as detailed by our laboratory [20], [21]. Briefly, mice were placed in a stereotaxic frame (Amscien Instruments, Richmond, VA, USA) and a 3.5 mm craniotomy was made in the right parietal bone midway between bregma and lambda with the medial edge 1 mm lateral to the midline, leaving the dura intact. Mice were impacted at 4.5 m/s with a 20 ms dwell time and 1 mm depression using a 3 mm diameter convex tip, mimicking a moderate TBI. Sham-operated mice underwent the identical surgical procedures, but were not impacted. The incision was closed with surgical staples and mice were allowed to recover. Throughout all procedures, body temperature was maintained at 37°C using a small animal temperature controller (Kopf Instruments, Tujunga, CA, USA). The NADPH oxidase inhibitor, apocynin (Sigma-Aldrich, 4 mg/kg) or saline were administered by intraperitoneal (IP) injections 20 min prior to TBI or 2 h after TBI. In addition, in some studies, a NOX2 competitive inhibitor, gp91ds–tat or its scrambled control peptide (Scr) (250 µg/mouse, synthesized by AnaSpec) were administered by IP injection 20 min prior to TBI.

### Assessment of Cerebral Edema

Brain water content (BWC), a sensitive measure of cerebral edema, was quantified using the wet-dry method, as detailed by our group [20], [22]. At 24 h post-injury, a time-point associated with significant edema formation after experimental TBI [20], [23], [24], BWC was estimated in 3 mm coronal sections of the ipsilateral cortex (or corresponding contralateral cortex), centered upon the impact site. Tissue was immediately weighed (wet weight), then dehydrated at 65°C. The sample was reweighed 48 h later to obtain a dry weight. The percentage of tissue water content was calculated using the following formula: BWC = [(wet weight–dried weight)/wet weight]×100%.

### Western Blot Analysis

Western blotting was performed as described in detail by our laboratory [25]. The pericontusional cerebral cortex was microdissected from brain tissue and immediately frozen in dry ice. Tissues samples were homogenized using a glass homogenizer with ice-cold homogenization buffer. Protein concentrations were determined by the Modified Lowry Protein Assay (Pierce, Rockford, ILL). Samples were subjected to gel electrophoresis, transferred to PVDF membranes, and Western blotted with an anti-β-Amyloid (sc-28365, Santa Cruz), anti-APP (#36-6900, Invitrogen) or anti-β-Actin (sc-81178, Santa Cruz) antibody. Bound proteins were visualized using the Odyssey Imaging System (LI-COR Bioscience, Lincoln, NB) and analyzed with the Image J analysis software (Version 1.30v; NIH, USA). Band densities were normalized to actin and expressed as fold changes of control animals. A Mean ± SE were calculated from the data from all the animals for graphical presentation and statistical comparison.

### Immunohistochemistry

DAB staining was performed using the VECTASTAIN Elite ABC Kit (Vector Laboratories, Inc., CA) as described previously by our laboratory [25]. Briefly, after blocking with normal goat/horse serum for 1 h, sections were incubated with the primary antibodies overnight at 4°C, followed by incubation with secondary biotinylated antibodies and ABC reagents for 1 h, separately. Color was developed with DAB reagent for 2–10 min. Images were captured using an AxioVision4Ac microscope system (Carl Zeiss, Germany).

### Confocal Microscopy and Image Analysis

Confocal analysis was performed as described previously by our laboratory [25]. Briefly, coronal brain sections (20 µm) were cut on a microtome after perfusion and cryoprotection. Staining was performed using a mouse anti-NeuN monoclonal antibody (1∶500, Chemicon, MA, USA), and the anti-CD11b monoclonal antibody (OX-42, 1∶500, Abcam) following the manufacturer's instruction. All the confocal images were captured on an LSM510 Meta confocal microscope and images were viewed using LSM510 Meta and Volocity 4.0 imaging software.

### Histology and Assessment of Surviving Cells

Coronal sections (25-µm thick) were collected throughout the entire dorsal hippocampus from animals sacrificed at 4 d after TBI, and every eighth section was collected and stained. Sections were stained with 0.1% (w/v) Cresyl violet for 5 min, dehydrated through graded concentrations of ethanol, and cleared in xylene. The slides were examined with a Zeiss Axioskop 40 (Zeiss, Germany), and the densities of surviving neurons in the pericontusional cerebral cortex and the medial CA1/CA3 pyramidal cell layer were counted on each section using the unbiased Stereologer System (Stereology Resource Center, Chester, MD, USA) [26]. The counting parameters utilized were the distance between counting frames (75 µm), the counting frame size (200×200 µm), the disector height (10 µm), and the guard-zone thickness (2 µm). Intact cells showing round nuclei but not condensed, pyknotic nuclei were counted as surviving cells. Means ± SE were calculated from the data in each group, and statistical analysis was performed as described below. The numbers of surviving cells were expressed as percentage of sham control.

### NADPH oxidase activity and superoxide production assay

NADPH oxidase activity was determined as described previously by our laboratory [27]. Briefly, assays were carried out in a final volume of 1 ml containing 50 mM Krebs-Ringer Phosphate buffer, pH 7.0, 1 mM EGTA, 150 mM sucrose, 0.5 mM lucigenin, 0.1 mM NADPH, and 50 µg tissue homogenate. Photoemissions, expressed in terms of relative light units (RLU), were measured every min for 5 min using a luminometer. Superoxide production was measured using a LumiMax Superoxide Anion Detection kit (Stratagene, La Jolla, CA) following the manufacturer's protocol. In brief, 50 µg sample proteins were suspended in 100 µl assay medium, and then mixed with 100 µl of reagent mixture containing 0.2 mM luminol, 0.25 mM enhancer. Light emissions at 30-sec intervals were recorded by a standard luminometer. All the values were standardized to the amount of protein and were calculated as RLU/µg protein/min. A Mean ± SE was calculated from the data collected in each group for graphical depiction.

### In situ detection of superoxide production

The production of superoxide (O^2−^) radicals was investigated using hydroethidine (HEt) (Molecular Probes, Invitrogen, CA) as described previously by our group and others [28], [29]. HEt (1 mg/ml in 200 µl PBS) was administered intravenously 30 min before ischemia. Fluorescent intensity of the oxidized HEt was measured on a confocal laser microscope using an excitation wavelength of 543 nm and the emission will be recorded at wavelengths between 560 and 590 nm.

### Statistical Analysis

Statistical analysis was performed using one-way analysis of variance (ANOVA) analysis, followed by Student-Newman-Keuls post-hoc tests to determine group differences. Statistical significance was accepted at the 95% confidence level (P<0.05). Data was expressed as mean ± standard error (SE).

## Results

### Temporal Induction of NADPH Oxidase Activation and O_2_^−^ Generation in the Brain Following TBI

**Figure 1:**
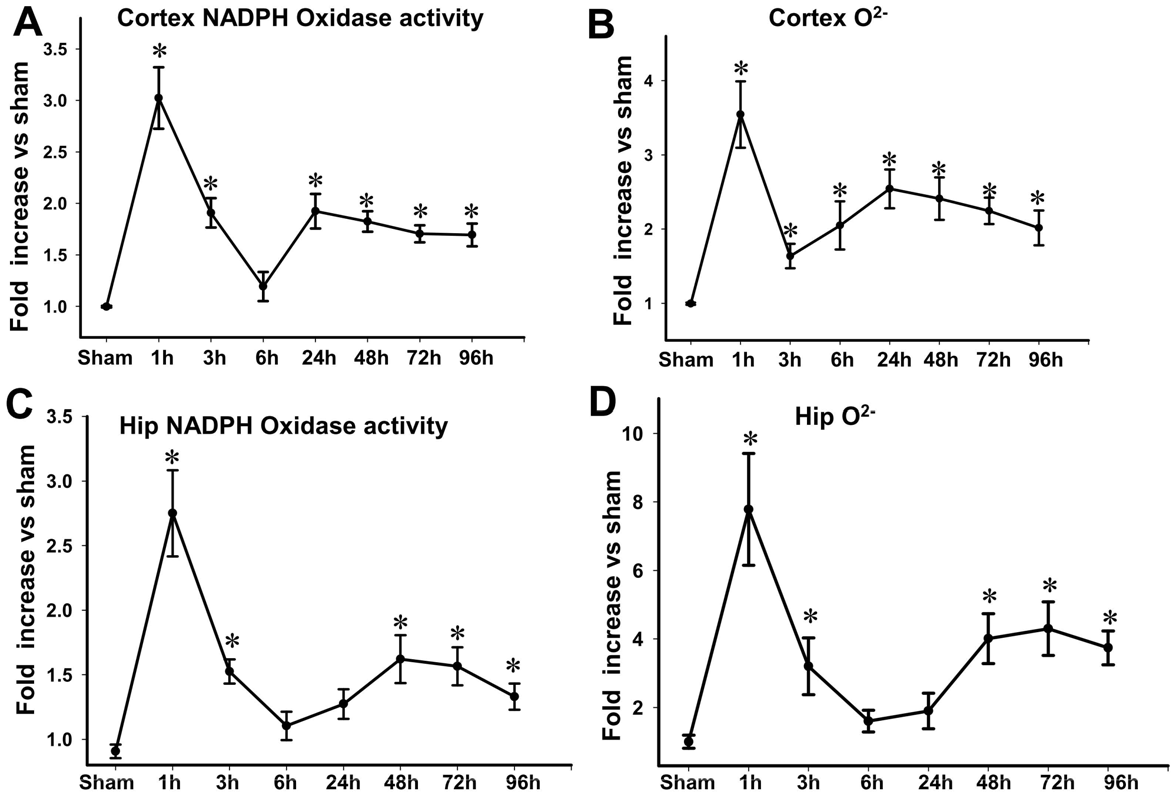
Temporal pattern of changes in NADPH oxidase activity and superoxide (O_2_^−^) levels in the cortex and hippocampus after TBI. Assays of NADPH Oxidase Activity (**A, C**) and O_2_^−^ production (**B, D**) were carried out using cerebral cortex and whole hippocampus (Hip) samples collected at the indicated times after TBI. Sham animals which did not undergo TBI were used as controls. Mean ± SE were calculated from the data collected in each group (n = 4) and expressed as fold changes vs. sham control. ^*^P<0.05 vs. sham in each panel.

**Figure 2:**
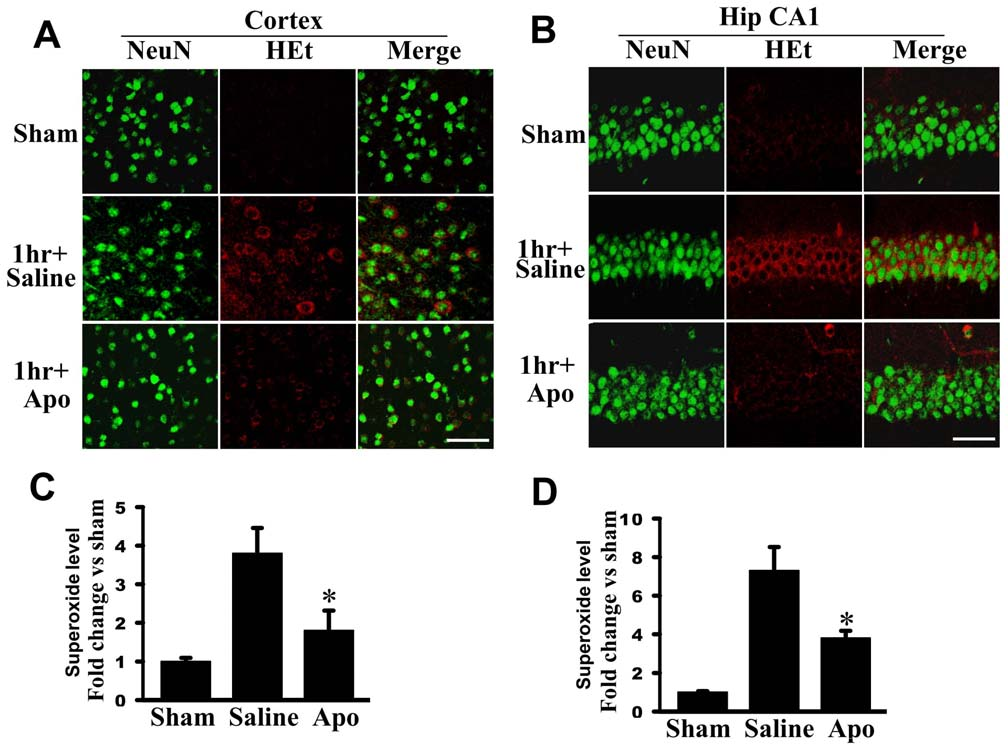
Effects of the NADPH oxidase inhibitor, apocynin on *in situ* superoxide levels (O_2_^−^) induced by TBI. **A–B:** Representative confocal microscopy of *in situ* O_2_^−^ (oxidized HEt, red) and NeuN (green) staining taken from cerebral cortex and medial hippocampal CA1 region (Hip CA1) at 1 h after TBI. **C–D:** Superoxide levels were measured using cerebral cortex and hippocampus samples, and shown as fold change vs. sham control (means±SE; n = 4 animals per group). Apocynin significantly attenuated O_2_^−^ generation compared with vehicle control. ^*^P<0.05 vs. saline in each group. Scale bars, 50 µm.

In initial studies, we first examined the temporal pattern of NADPH oxidase activation and O_2_^−^ generation in the adult male mouse cerebral cortex and whole hippocampus samples following moderate TBI induced by controlled cortical contusion. As shown in **Fig. 1**, there was rapid, robust elevation of NADPH oxidase activity and O_2_^−^ levels in the cerebral cortex and hippocampus at 1 h after TBI. This rapid elevation of NADPH oxidase activity and O_2_^−^ levels was followed by a fall to lower but still elevated levels at 3–6 h after TBI. Interestingly, a second significant elevation of NADPH oxidase activity and O_2_^−^ levels was also observed from 24 h–96 h after TBI. In the majority of the subsequent studies, we chose to use the 1 h time-point for further analysis, as this was when peak NADPH oxidase activity and O_2_^−^ levels were observed. We next sought to confirm *in situ* elevation of O_2_^−^ in the cortex and hippocampus by utilizing the hydroethidine (HEt) method, in which HEt is selectively oxidized by O_2_^−^ to yield a fluorescent signal that is easily detectable in brain sections by confocal microscopy. We also used immunohistochemistry for the neuronal marker, NeuN, so as to determine if changes in O_2_^−^ generation occurs in neurons. As shown in **Fig. 2** *in situ* O_2_^−^ levels, as measured by oxidized HEt fluorescent signal, increased markedly at 1 h after TBI in the cortex (**Fig. 2A**) and hippocampal CA1 region (**Fig. 2B**) in saline-treated animals as compared to sham control animals. Furthermore, the HEt fluorescent signal was strongly colocalized in cortical and hippocampal neurons, as evidenced by colocalization with the neuronal marker NeuN, suggesting that neurons are major sources of O_2_^−^ generation in the cortex and hippocampus in the initial early period following TBI (**Fig. 2A&B**). It is likely that microglia are the source of superoxide at later time-points; however, this could not be confirmed using the HEt method as the elevation of superoxide was much lower at 24–96 h as compared to 1 h post TBI and the sensitivity of the HEt method was too low to allow detection.

### Role of NADPH Oxidase in O_2_^−^ Generation and Oxidative Damage in the Brain Following TBI

**Figure 3:**
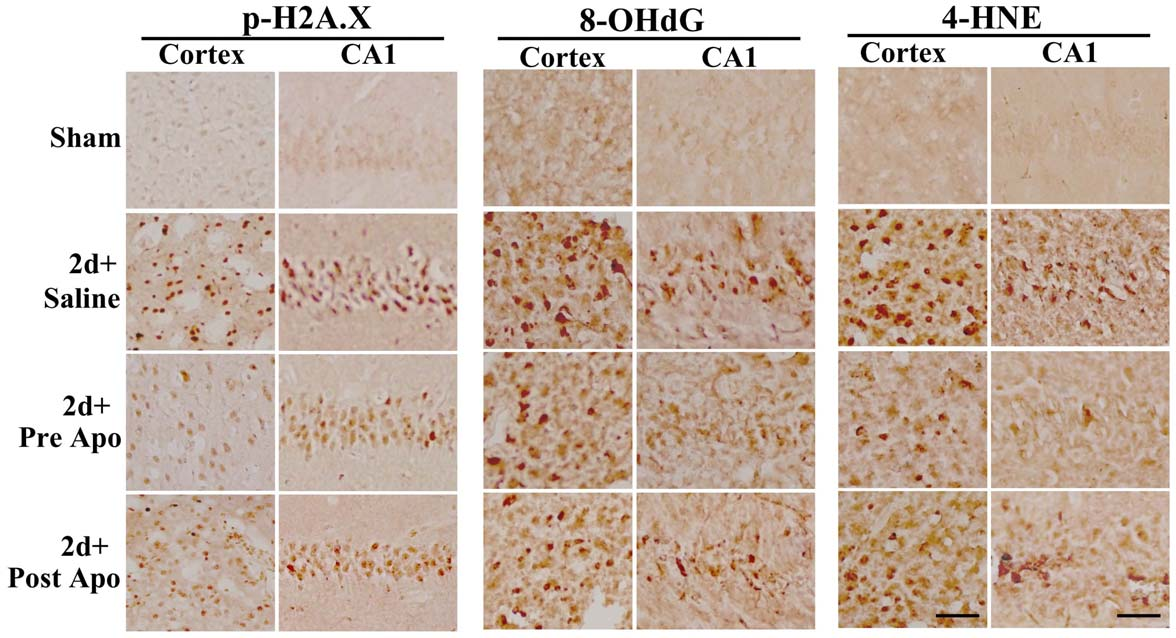
Effect of apocynin on TBI-induced oxidative damage in the cerebral cortex and hippocampal CA1 region. Representative DAB immunostaining for markers of oxidative damage for lipid peroxidation (4-HNE), DNA damage (8-OHdG) and histones (phospho-H2A.X) 2 days following TBI. Note that both pre- and post-treatment of apocynin markedly decreased 4-HNE, 8-OHdG and phohpho-H2A.X staining in cerebral cortex and hippocampus CA1. The results are representative of 4–5 animals per group. Scale bars, 50 µm.

We next examined whether the increase in O_2_^−^ generation in cortical and hippocampal neurons following TBI was due to activation of NADPH oxidase. This was determined by examining the effect of pretreatment with the NADPH oxidase inhibitor, apocynin (4 mg/kg ip, 20 min prior to TBI) on O_2_^−^ generation in cortical and hippocampal neurons following TBI. As shown in representative photomicrographs in **Fig. 2A–B**, pretreatment with apocynin markedly attenuated generation of O_2_^−^ in the cortical and hippocampal neurons at 1 h after TBI as compared to the saline-treated group, suggesting that NADPH oxidase plays a critical role in O_2_^−^ production in neurons following TBI. **Fig. 2C–D** shows the mean values and statistical analysis of O_2_^−^ generation (as measured by using cerebral cortex and hippocampal protein samples) in all animals, demonstrating that there is an approximate 3-fold and 7-fold increase in O_2_^−^ levels in the cortex and hippocampus, respectively, in saline-treated animals at 1 h after TBI as compared to sham controls, and that pretreatment with the NADPH oxidase inhibitor, apocynin markedly attenuated the induction of O_2_^−^ following TBI. These findings suggest that NADPH oxidase has a major role in the robust O_2_^−^ generation observed in the cortex and hippocampus in the early period following TBI. We thus next examined whether inhibition of NADPH oxidase via administration of apocynin leads to a decrease in oxidative damage in the brain following TBI. To accomplish this aim, brain sections were collected at 2 days after TBI and immunohistochemistry was performed for several well characterized markers of oxidative damage, including 4-HNE (4-hydroxynonenal), 8-OHdG (8-hydroxydeoxyguanosine), and p-H2AX (phospho-histone), which are markers of lipid peroxidation, DNA damage and oxidative histone phosphorylation, respectively. The results show robust increases in immunostaining intensity for all three oxidative stress markers in the cortex and hippocampal CA1 region in the saline-treated (TBI) group as compared to sham controls (**Fig. 3**). Interestingly, both pre- and post-treatment with the NADPH oxidase inhibitor, apocynin markedly attenuated the oxidative stress damage following TBI, as indicated by a strong attenuation of 4-HNE, 8-OHdG and p-H2AX immunostaining in the cortex and hippocampal CA1 region as compared to saline-treated (TBI) controls (**Fig. 3**). The results suggest an important role for NADPH oxidase in oxidative stress damage to the brain following TBI.

### Role of NADPH Oxidase in Microglia Activation Following TBI

**Figure 4:**
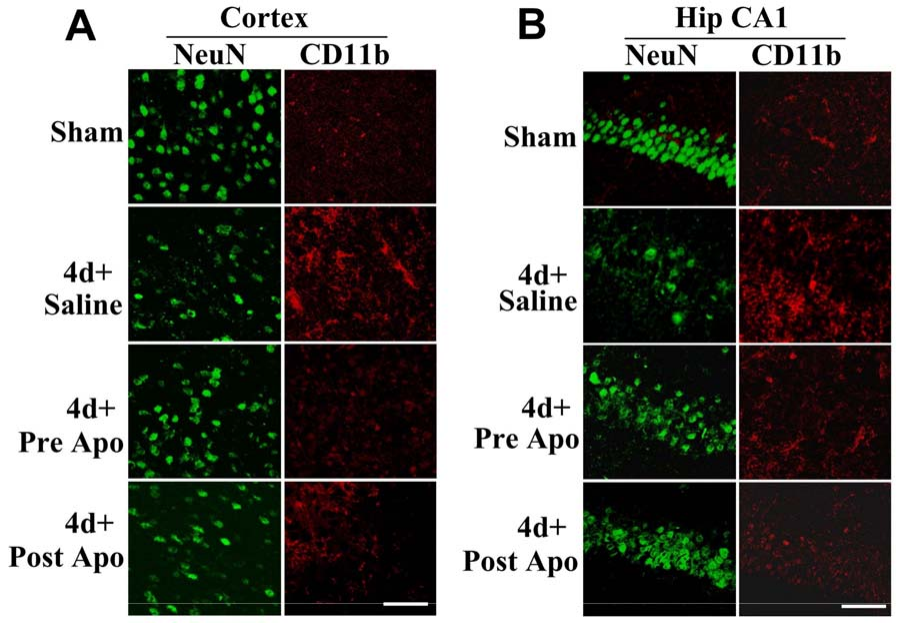
Apocynin increases neuronal density and attenuates microglia activation following TBI. Coronal brain sections containing cerebral cortex (**A**) and hippocampal CA1 region (**B**) were subjected to immunofluorescent staining with anti-NeuN (green) and anti-CD11b (red) antibodies. Representative images indicate that both pre- and post-treatment of apocynin markedly increased neuronal density and reduced microglial activation at 4 d after TBI. The results are representative of 4–5 animals per group. Scale bars, 50 µm.

It is well known that microglial activation occurs several days after TBI and plays a role in inflammation and pathology of TBI. We thus examined whether activation of NADPH oxidase has a critical role in microglial activation following TBI by assessing the effect of administration of apocynin upon microglia activation following TBI. To access microglial activation, we performed immunohistochemistry on brain sections at 4 d after TBI for the microglial activation marker, CD11b. We also performed immunostaining with the neuronal marker, NeuN in order to assess apocynin effects upon preservation of neuronal density. As shown in **Fig. 4 A&B**, sham animals showed robust NeuN immunostaining and low CD11b immunostaining in the cortex and hippocampal CA1 region, indicating healthy neurons and little microglial activation in the non-injured state. In contrast, saline-treated animals examined at 4 d after TBI showed clear reduction of immunostaining intensity for the neuronal marker NeuN, and robust immunostaining for the microglial activation marker, CD11b in the cortex and hippocampal CA1 region, as compared to the sham control (**Fig. 4A&B**). Intriguingly, both pre- and post-treatment of apocynin markedly attenuated CD11b immunostaining in the cortex and hippocampal CA1 region at 4 d after TBI, suggesting a role for NADPH oxidase activation in microglial activation following TBI. In addition, apocynin-pretreated animals showed enhanced NeuN immunostaining in both the cerebral cortex and hippocampal CA1 region at 4 d after TBI, suggesting enhanced preservation of neurons following TBI.

### NADPH Oxidase Activation Mediates β-Amyloid Induction in the Brain Following TBI

**Figure 5:**
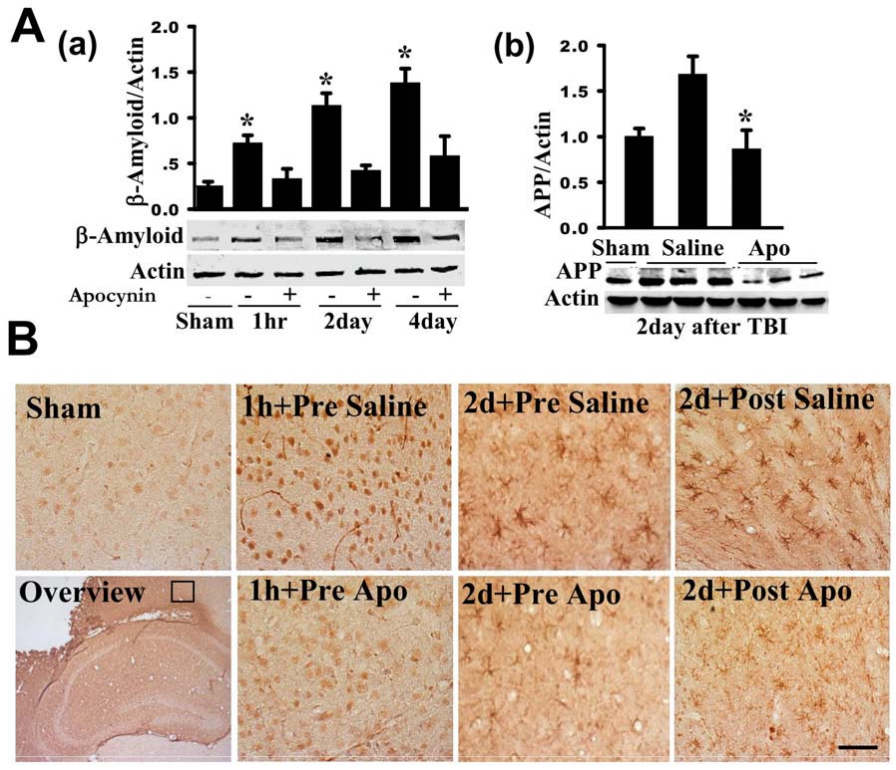
Effects of apocynin on levels of β-amyloid and amyloid precursor protein (APP) in the cortex and hippocampus following TBI. **A(a,b):** At the indicated time points after TBI, protein samples from pericontusional cerebral cortex were analyzed by Western blotting with an anti-β-amyloid or anti-APP antibody. β-Actin was used as loading control. β-amyloid and APP protein levels were significantly increased in the cerebral cortex at the examined time-points, and apocynin pretreatment significantly attenuated the elevation. Data are mean±SE (n = 4 in each group). ^*^P<0.05 vs. vehicle control. **B:** Representative DAB immunostaining of β-amyloid on coronal brain sections from sham and brain injured mice at the indicated time points following TBI. Note that apocynin markedly attenuated TBI induced increase of β-amyloid levels mainly in the neuronal soma (at 1 h) and microglia like cells (at 2 d). Both pre- and post-treatment of apocynin strongly attenuated the β-amyloid induction. The results are representative of 4–5 animals per group. Scale bar, 50 µm.

It has been previously shown that TBI is associated with an elevation of β-amyloid levels in the brain, which may explain an increased risk for cognitive decline and dementia in TBI patients [30], [31]. We therefore examined β-amyloid induction in the brain at various time-points following TBI and assessed the potential role of NADPH oxidase activation in β-amyloid induction via use of the NADPH oxidase inhibitor, apocynin. As shown in **Fig. 5A(a)**, Western blot analysis revealed that β-amyloid protein levels are significantly increased in the cerebral cortex at all time-points examined (1 h, 2 d and 4 d after TBI) as compared to sham controls. Interestingly, apocynin pretreatment significantly attenuated the elevation of β-amyloid protein levels in the cortex at all time-points examined following TBI (**Fig. 5A**), suggesting that NADPH oxidase activation is important for the induction of β-amyloid. **Fig. 5A(b)** shows a similar pattern of change for amyloid precursor protein (APP) when examined at the 2day time-point, with TBI (saline) showing increased levels of APP in the cortex and treatment with apocynin preventing the increase of APP. To provide more information on potential cell types and organelles in which the β-amyloid changes occur, we utilized immunohistochemistry. As shown in **Fig. 5B**, sham animals showed very little β-amyloid immunostaining in the cortex. In contrast, saline treated (TBI) animals showed robust β-amyloid immunostaining in the cortex at the 1 h time-point after TBI, with the staining appearing to be in neuronal soma and axons, at least from morphological appearance (e.g. immunostaining in round cells and in long fiber like processes). At 2 d after TBI, many cells in the cortex of saline-treated (TBI) mice that were β-amyloid-positive had the morphological appearance of microglia. Both pre- and post-treatment of apocynin caused a marked attenuation of β-amyloid immunostaining intensity in the cortex (**Fig. 5B**) at 2 d following TBI. We should add that β-amyloid immunostaining was also observed in the hippocampal CA1 region, but at lower levels than the cortex, and that apocynin similarly decreased the β-amyloid immunostaining intensity in the hippocampal CA1 region as it did in the cortex (data not shown).

### Administration of the NADPH Oxidase Inhibitor, Apocynin, Provides Significant Neuroprotection Against TBI

**Figure 6:**
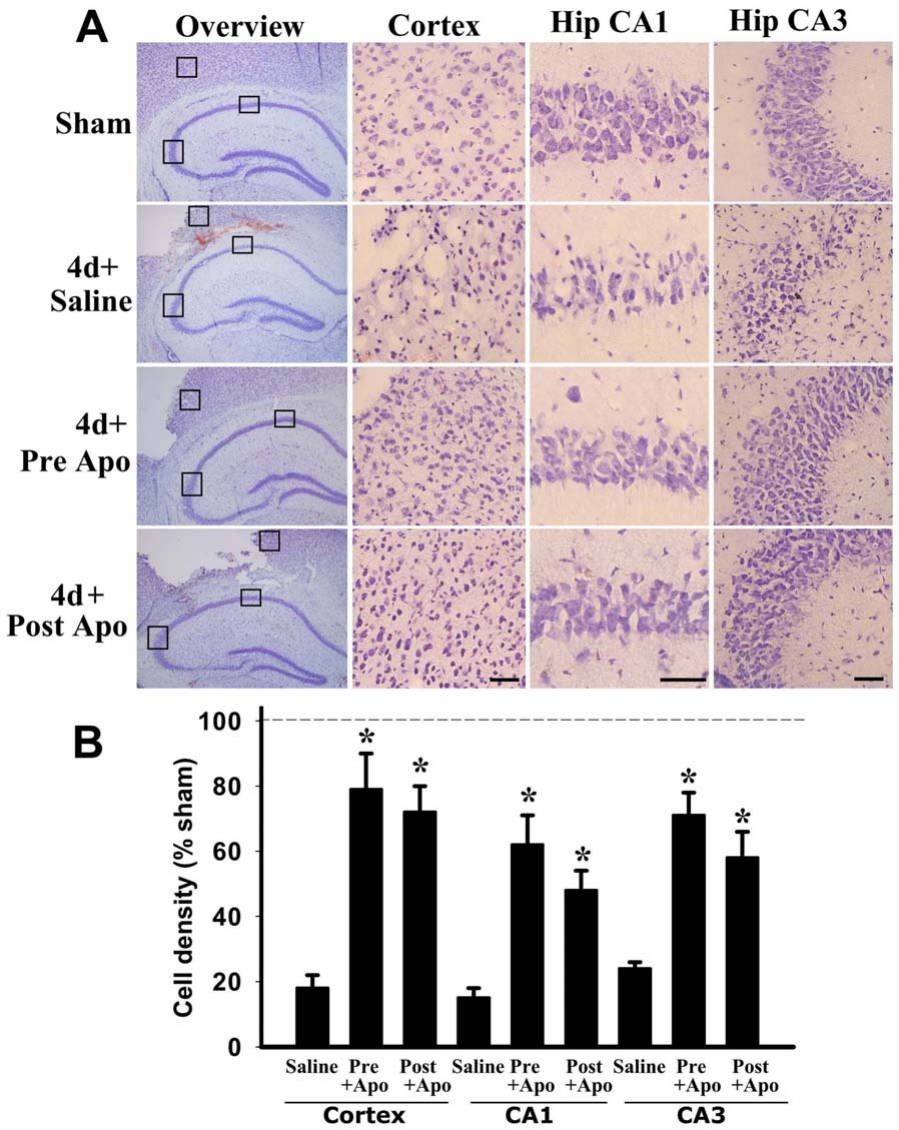
Neuroprotective effects of apocynin in the cerebral cortex and hippocampus(CA1 and CA3 regions) following TBI. **A:** Representative Cresyl violet staining on day 4 after TBI from sham, saline- and apocynin-pre/post treated mice. Boxed areas in left column are shown at higher magnification in right columns. **B:** Quantitative analysis of the numbers of surviving cells expressed as % of sham control. Intact cells showing round nuclei but not condensed, pyknotic nuclei were counted as surviving cells. Data are mean ± SE (n = 5–7) and a typical experiment is presented. ^*^P<0.01 vs. saline in each group. Scale bars, 50 µm.

Since apocynin inhibited ROS production, oxidative damage, microglial activation and β-amyloid/APP induction in the brain following TBI, we next examined whether apocynin exerts significant neuroprotection against TBI. Apocynin (4 mg/kg ip) was administered 20 min before TBI, and cell death in the cerebral cortex and hippocampus was examined 4 days after TBI using the cresyl violet staining procedure. **Figure 6A** presents representative photomicrographs of the cresyl violet staining results in the cerebral cortex and hippocampal CA1 and CA3 regions at 4 d after TBI. The cresyl violet staining results show that while cortical and hippocampal cells in sham animals displayed round and pale stained nuclei typical of normal healthy cells; cresyl violet-stained cortical and hippocampal cells in saline-treated (TBI) animals had a shrunken morphology with pyknotic nuclei, indicating many dead or dying cells in the cortex and hippocampal CA1 and CA3 regions. Apocynin pre-administration dramatically attenuated TBI-induced neuronal cell death in the cerebral cortex and hippocampus at 4 days after TBI, as determined by cresyl violet staining, which revealed less damaged or dead cells and more healthier, normal cells as compared to the saline-treated (TBI) control group. Unbiased stereological analysis was used to determine cell density in the cortex and hippocampus following TBI. As shown in **Fig. 6B**, the results revealed that saline-treated (TBI) animals have very low cell density as compared to sham controls in the cerebral cortex and hippocampal CA1 and CA3 regions at 4 d after TBI. Additionally, apocynin pretreatment resulted in a preserved cortical and hippocampal cell density – e.g. up to 60–70% of the sham controls, indicating a strong and significant neuroprotective effect of apocynin pretreatment. Interestingly, apocynin post-administration at 2 h after TBI also significantly attenuated the delayed neuronal cell death. This suggests that NADPH oxidase activation and superoxide elevations *after* the peak induction at 1 h play a major role in neuronal cell death following TBI.

### Evidence that the NOX2 isoform of NADPH Oxidase Plays a Critical Role in Neuronal Damage Following TBI

**Figure 7:**
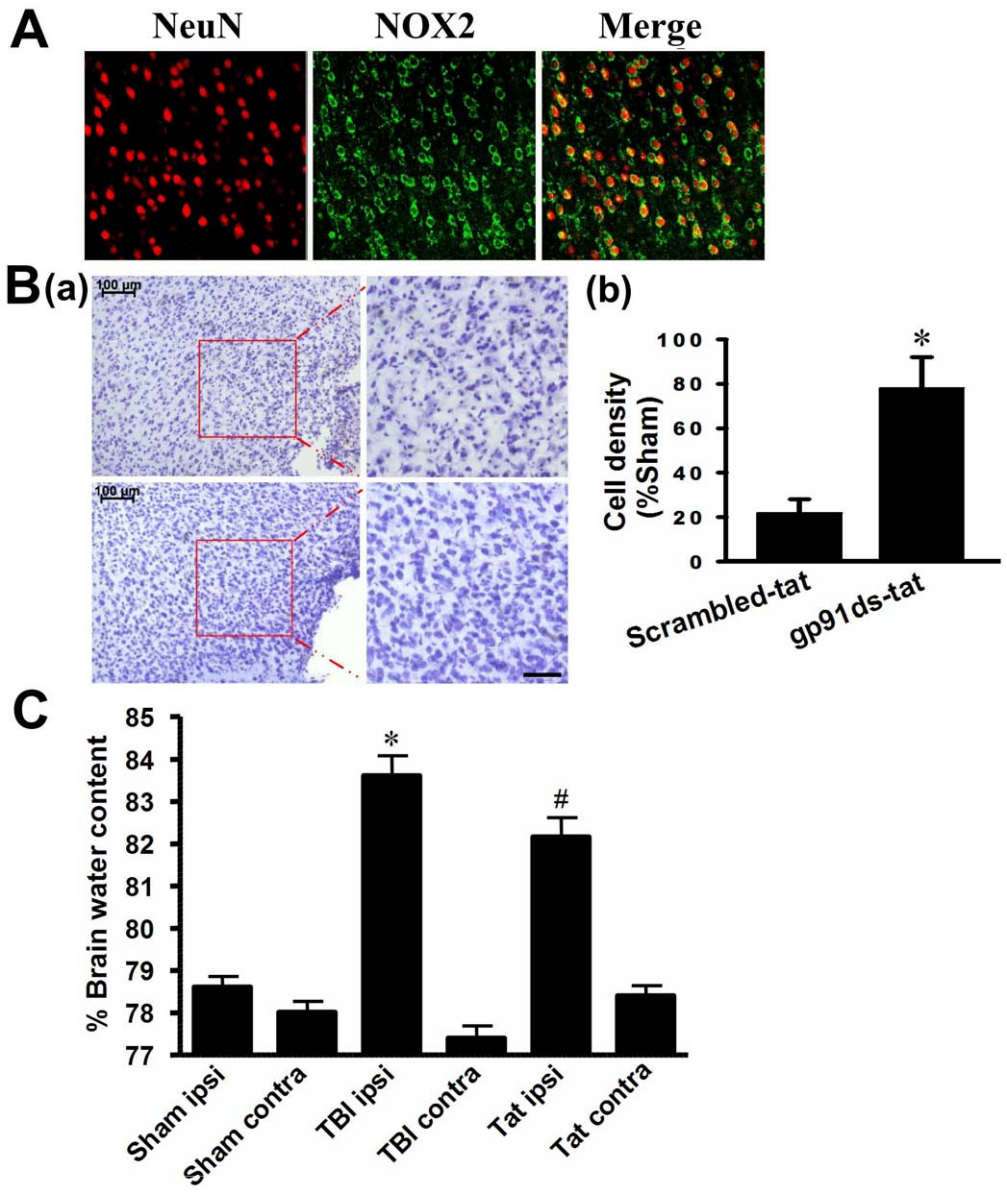
Critical Role of the NOX2 NADPH Oxidase isoform in neuronal damage and brain edema following TBI. **A:** Confocal images show colocalization of the NOX2 NADPH oxidase isoform and NeuN in cerebral cortex at 1 h after TBI. **B:** Cresyl Violet staining and cell-counting studies showing neuroprotective effects of the NOX2 NADPH oxidase inhibitor, gp91ds-tat at 4 days after TBI. Note the enhanced neuronal density in the cerebral cortex in gp91ds-tat treated mice as compared to the scrambled-tat peptide control (Scr)-treated mice. Data are expressed as mean±SE, n = 5–7 in each group. ^*^P<0.05 vs. scrambled-tat control. Scale bar, 50 µm. **C:** Brain water content was measured in the ipsilateral hemisphere at 1 day following TBI. Pretreatment with gp91ds-tat significantly reduced brain edema. Data are mean±SE (n = 12). ^*^P<0.05 vs. scrambled-tat control.

We next examine the role of the major NOX2 isoform of NADPH oxidase in TBI damage. **Fig. 7A** shows the results of double immunohistochemistry for NOX2 and the neuronal marker, NeuN in the cerebral cortex at 1 h after TBI. The results show that NOX2 is expressed in the extranuclear membrane region of cortical cells 1 h after TBI and is highly colocalized in neurons as evidenced by colocalization with NeuN. To determine the role of NOX2 in TBI damage in the cortex, we administered a competitive NOX2 inhibitor, gp91ds-tat, which is a 9 amino acid peptide inhibitor that binds the p47Phox binding site on NOX2, preventing activation of NOX2 [32]. A scrambled tat peptide was also administered as a control. As shown in **Fig. 7B (a–b)**, pretreatment with gp91ds-tat resulted in significant neuroprotection against TBI as evidenced by enhanced neuronal density in the cerebral cortex as compared to the scrambled-tat peptide control. In addition, we examined the ability of the NOX2 inhibitor to reduce brain edema following TBI. As shown in **Fig. 7C**, TBI induced a significant increase in brain edema in the ipsilateral hemisphere as compared to the contralateral hemisphere and sham controls. Pretreatment with gp91ds-tat significantly reduced brain edema in the ipsilateral hemisphere following TBI.

## Discussion

ROS generation and oxidative stress has been implicated to contribute significantly to neuronal cell death and functional impairments following TBI [9], [10], [33]. The current study adds to our understanding regarding the source of the ROS by demonstrating that the membrane, via activation of the enzyme NADPH oxidase, contributes significantly to generation of O_2_^−^ in the cerebral cortex and hippocampus. There are at least five different isoforms of NADPH oxidase identified to date, termed NOX 1–5 [14]. The NOX2 isoform has been shown to be highly expressed in the cortex and hippocampus and to be involved in oxidative damage following focal and global cerebral ischemia [15], [27], [34]–[36].

In our study, two peaks of NADPH oxidase activation were observed at 1 h and 24–96 h after TBI, which paralleled elevations of O_2_^−^. The secondary peak of NADPH oxidase may have the greater role in neuronal cell death, as apocynin post-treatment after the first peak of NADPH oxidase (e.g. 2 h after TBI) still exerted significant neuroprotection, which was statistically not different from pretreatment (although mean values for neuronal cell density were 5–15% lower in the post-treatment group). That NADPH oxidase activation is responsible for the O_2_^−^ elevations observed in our study was suggested by the fact that administration of the NADPH oxidase inhibitor, apocynin strongly attenuated the elevation of O_2_^−^. O_2_^−^ is well known to be metabolized to highly toxic free radicals such as hydroxyl ion and peroxynitrite, which cause oxidative damage to cells and neurons [37]. Our study showed that NADPH oxidase activation plays an important role in oxidative damage to neurons following TBI, as pre- or post-treatment with the NADPH oxidase inhibitor, apocynin significantly reduced oxidative damage in the cortex and hippocampus, as evidenced by assessment of oxidative stress markers of lipid peroxidation and DNA oxidative damage, and significant neuroprotection against TBI. Intriguingly, apocynin has also been shown to reduce ischemic injury and improve outcome in mouse stroke models [35], [38], [39], suggesting that NADPH oxidase activation also has a pathological role in ischemic type injury in addition to concussion injury. Furthermore, the ability of apocynin to reduce neuronal cell death, neurological impairment and mortality in the stroke studies was lost when it was administered in NOX2 knockout mice, which strongly suggests that the beneficial neural effects of apocynin are due specifically to inhibition of NOX2 NADPH oxidase [35].

Our study implicates NOX2 NADPH oxidase as having a significant role in TBI, as dual immunohistochemistry studies revealed that NOX2 is highly colocalized in neurons at 1 h after TBI. Furthermore, pretreatment with the specific NOX2 inhibitor, gp91ds-tat significantly attenuated neuronal damage and edema following TBI. It should be pointed out that a recent study using NOX2 mutant knockout mice found that TBI damage to the brain was likewise significantly attenuated in NOX2 knockout mice as compared to wild type mice [19]. In that study, which examined only late time-points (24–48 h) after TBI, the authors concluded that NOX2 activation in microglia at 24–48 h contributed significantly to neuronal damage following TBI. Our study examined both early and late time-points after TBI and found that there are two peaks of NADPH oxidase activation and O_2_^−^ elevation, an early peak at 1 h and a delayed peak at 24–96 h after TBI. The first peak appears to be of neuronal origin as colocalization studies showed strong colocalization of NOX2 and *in situ* O_2_^−^ (HEt) signal in neurons at 1 h after TBI. The cellular source for the NADPH oxidation and O_2_^−^ elevation at 24–96 h is unclear, but our study showed enhanced microglial activation that paralleled the NADPH oxidase activation and enhanced O_2_^−^ levels at 24–96 hr. This observation suggests that enhanced microglia activation likely contributes to the delayed secondary elevation of NADPH oxidase activation and O_2_^−^ production after TBI. In support of this possibility, NOX2 knockout mice have been reported to have significantly reduced generation of O_2_^−^ in microglia at 24–48 h after TBI [19]. From a functional standpoint, microglial activation has been implicated to play a role in mediating the inflammation that occurs after TBI and contributing to neuronal damage through release of inflammatory cytokines [40], [41]. Microglia have also been shown to enhance beta-amyloid–induced neurotoxicity [42]. Thus, the attenuation of microglia activation following NADPH oxidase inhibition could potentially reduce inflammation and β-amyloid-induced neurotoxicity, and facilitate neuronal survival following TBI. In support of this suggestion, post-treatment with apocynin at 2 h after TBI was strongly neuroprotective. Previous work has shown that TBI is also associated with a significantly enhanced risk of cognitive decline and dementia [31], [43]. Intriguingly, TBI has been demonstrated to induce significant elevations of the AD proteins, APP and β-amyloid protein in the regions adjacent to the site of injury [30], [44]. Our study confirms elevation of APP and β-amyloid in the brain following TBI, and extends these observations by providing evidence that NADPH oxidase activation is critical for the induction of APP and β-amyloid.

In conclusion, the results of the current study demonstrate that the membrane, via NADPH oxidase activation can contribute significantly to ROS generation, oxidative stress damage, and neuronal cell death following TBI. The study also suggests that targeting NADPH oxidase for inhibition via use of specific NADPH oxidase inhibitors may have clinical efficacy in TBI.
